# Diagnosis and treatment of intractable idiopathic orofacial pain with attention-deficit/hyperactivity disorder

**DOI:** 10.1038/s41598-023-28931-3

**Published:** 2023-01-30

**Authors:** Satoshi Kasahara, Kaori Takahashi, Ko Matsudaira, Naoko Sato, Ken-ichi Fukuda, Akira Toyofuku, Tatsuya Yoshikawa, Yuichi Kato, Shin-Ichi Niwa, Kanji Uchida

**Affiliations:** 1 Department of Anesthesiology and Pain Relief Center, The University of Tokyo Hospital, 7-3-1 Hongo, Bunkyo-ku, Tokyo, 113-8655 Japan; 2 Department of Pain Medicine, Fukushima Medical University School of Medicine, 1 Hikarigaoka, Fukushima, Fukushima 960-1295 Japan; 3 Department of Dental Anesthesiology, Tokyo Dental College, 2-9-18, Misakicho, Chiyoda-ku, Tokyo, 101-0061 Japan; 4 Department of Medical Research and Management for Musculoskeletal Pain, 22nd Century Medical and Research Center, The University of Tokyo Hospital, 7-3-1 Hongo, Bunkyo-ku, Tokyo, 113-8655 Japan; 5 Nursing Department, The University of Tokyo Hospital, 7-3-1 Hongo, Bunkyo-ku, Tokyo, 113-8655 Japan; 6 Division of Special Needs Dentistry and Orofacial Pain, Tokyo Dental College, 2-9-18, Kanda-misakicho, Chiyoda-ku, Tokyo, 101-0061 Japan; 7 Department of Psychosomatic Dentistry, Graduate School of Medical and Dental Sciences, Tokyo Medical and Dental University, 1-5-45 Yushima, Bunkyo-ku, Tokyo, 113-8549 Japan; 8 Luxia Ginza Dental Clinic, 3-7-13 Ginza, Chuo-ku, Tokyo, 104-0061 Japan; 9 Department of Pediatric Dentistry, School of Life Dentistry at Tokyo, Nippon Dental University, 1-9-20 Fujimi, Chiyoda-ku, Tokyo, 102-8159 Japan; 10 Department of Psychiatry, Aizu Medical Center, Fukushima Medical University, 21-2 Maeda, Yazawa, Kawahigashi-Machi, Aizuwakamatsu, Fukushima 969-3492 Japan

**Keywords:** Dentistry, Drug regulation, Chronic pain, Developmental disorders

## Abstract

Attention-deficit/hyperactivity disorder (ADHD) has been reported to be associated with primary chronic pain syndromes, such as fibromyalgia, migraine, and chronic low back pain. Although idiopathic orofacial pain (IOP) is classified as burning mouth syndrome or persistent idiopathic facial or dentoalveolar pain and as a primary chronic pain, the association between IOP and ADHD has not been investigated. This retrospective cohort study investigated the severity of ADHD symptoms measured using the ADHD scale and the effects of treatment using ADHD drugs and the dopamine system stabilizer aripiprazole. The participants were 25 consecutive patients with refractory IOP referred to a psychiatrist and diagnosed with coexisting ADHD according to the Diagnostic and Statistical Manual of Mental Disorders-5. The ADHD scale scores were higher in patients with intractable IOP than those in the general population. Pharmacotherapy used in this study led to clinically significant improvements in pain, anxiety/depression, and pain catastrophizing. Intractable IOP and ADHD were shown to be associated. In the future, screening and pharmacotherapy for ADHD should be considered in the treatment of intractable IOP.

## Introduction

According to the International Classification of Orofacial Pain (ICOP), 1st edition, idiopathic orofacial pain (IOP) is defined as unilateral or bilateral intraoral or facial pain of unknown etiology in the distributions of one or more branches of the trigeminal nerve(s)^1^. Pain is usually persistent, moderately intense, non-localized, and described as dull, oppressive, or burning. IOP is further classified as burning mouth syndrome (BMS), persistent idiopathic facial pain (PIFP), or persistent idiopathic dentoalveolar pain (PIDAP). BMS is characterized by bilateral, superficial, persistent pain in the oral mucosa, with a predilection for the lingual apex, lingual border, and palate; it occurs more commonly in women than in men, especially after menopause^2^. PIFP is persistent facial and/or oral pain of unclear localization with various symptoms and female predilection^3^. PIDAP is a deep pain in the teeth and alveolar region with well-defined localization, relatively low age of onset, and little gender difference; 70–83% of incidences are triggered by dental treatment^4^.

The incidences of BMS and PIFP, including PIDAP, in the general population, are 0.1–3.9%^5^ and 0.03%^6^, respectively. However, in clinical settings, such as oral-facial pain clinics, the incidence is reportedly 7.72% for BMS^7^, 10–21% for PIFP at orofacial pain clinics^3^, and 2.1% for PIDAP at tertiary medical centers^4^.

The pathophysiology of all three disorders is unclear; however, psychosomatic factors and central nervous system dysfunction are reportedly involved^8^, and tricyclic antidepressants (TCAs)^9^ and cognitive behavioral therapy^10^ are likely to be effective. IOP is a difficult disease to treat as its response to various therapies is inconsistent, with limited efficacy. IOP has a spontaneous remission rate of 3–4% even 5–6 years after the diagnosis is confirmed^11^, and patients with IOP may visit several dentists and doctors to understand the cause of their pain and for effective treatment. Although dental procedures may provide temporary improvement in the symptoms, they may subsequently reoccur in other teeth and the original locations, triggering a vicious cycle of further invasive procedures at the patient's request and despite the dentist's good intentions. Over the course of this process, the patient may lose multiple healthy teeth and experience pain in the entire orofacial region. Therefore, clinicians have great difficulty in managing patients with IOP^12^.

It was reported that 41.3% of patients with IOP had a history of a mental disorder before the onset of pain, approximately half of the patients had long-term psychiatric disorders, and one-third had a psychiatric comorbidity at the time the survey was conducted, indicating that mental disorders show a chronic, long-term course. The most common psychiatric comorbidities were major depression, anxiety disorder, and Diagnostic and Statistical Manual of Mental Disorders-5 (DSM-5) anxious and fearful Cluster C personality disorders (avoidant, dependent, and obsessive–compulsive personality disorders)^8^. In addition, the majority of the patients also had one or more other chronic pain symptoms, and almost all patients had other pain symptoms when they developed orofacial pain, suggesting that the three IOP disorders have common vulnerabilities to chronic pain and psychiatric disorders. IOP disorders are reportedly caused by dysfunction of the dopaminergic system, and it has been proposed that they are on the same neuroplastic pain spectrum, only with different degrees of disability^8,13^.

Attention-deficit/hyperactivity disorder (ADHD), a developmental disorder, is also reportedly associated with chronic pain. ADHD is characterized by attention deficit, hyperactivity, and/or impulsivity, which persists for a relatively long period from childhood to adulthood and causes dysfunction in daily living^14^. The pathophysiology of ADHD is dysfunction of the dopaminergic and noradrenergic nervous systems. Psychostimulants that enhance dopamine neurotransmission, selective noradrenaline reuptake inhibitors, noradrenergic alpha-2 receptor agonists^15^, and dopamine system stabilizers^16^ can improve ADHD symptoms. Previous studies suggested that ADHD is associated with fibromyalgia^17–22^, migraine^23^, and chronic low back pain^24,25^. These pain disorders are classified as primary pain syndromes in the International Classification of Disease, Eleventh Revision^26^, suggesting that ADHD is likely to coexist with chronic primary pain. IOP is also classified as a type of primary chronic pain; however, although no previous reports on a connection between IOP and ADHD are available, similar to IOP, patients with ADHD are more likely to experience depression, anxiety disorders, and DSM-5 anxious and fearful Cluster C personality disorders than the general population^27^. As the neuropathology of ADHD is also thought to be caused by dopaminergic system dysfunction^28^, IOP and ADHD may coexist as they share common origins.

Herein, we conducted a retrospective cohort study of patients with intractable IOP referred to a psychiatrist by specialists in psychosomatic dental care due to difficulty in managing their symptoms to delineate the characteristics of refractory patients. This study focused on the severity of ADHD symptoms measured using the ADHD scale, and the effectiveness of treatment using ADHD drugs and the dopamine system stabilizer aripiprazole (APZ) in intractable IOP.

## Methods

### Study design, settings, and patients

This cohort study retrospectively investigated the ADHD scale scores and ADHD diagnoses in patients with refractory IOP during the initial visit to examine whether there is an association between intractable IOP and ADHD. The severity of ADHD symptoms was assessed using Conners' Adult ADHD Rating Scale (CAARS), and ADHD was diagnosed according to the DSM-5. The attending psychiatrist (S.K.) routinely performs the CAARS evaluation at the patient's first visit.

We reviewed the clinical assessments of pain, mood, and catastrophic thinking conducted by the psychiatrist in every routine session, along with the medication algorithms followed by the psychiatrist in his usual practice, and evaluated the results of the practice through longitudinal analysis. Pain intensity was assessed using the Numerical Rating Scale (NRS), mood using the Hospital Anxiety and Depression Scale (HADS), and catastrophic thinking using the Pain Catastrophizing Scale (PCS), and compared before and after treatment. Dr. SK routinely evaluated patients using the "Pain NRS," the "HADS", and the "PCS”, and provided feedback on the scale scores to the patients at each visit.

Thirty consecutive patients with refractory IOP were referred to a psychiatrist (S.K.) at the Pain Center of our university hospital between May 2016 and March 2020 with suspected somatic symptom disorder after it was determined that treatment would be difficult by a specialist in psychosomatic dentistry at a tertiary medical center. The diagnosis of IOP was made by a specialist in psychosomatic dentistry who referred the patient. The definition of intractable IOP is undefined; therefore, we defined this condition as a strong and lasting IOP that did not respond to psychoeducation, psychotherapy, and the use of three or more medications (e.g., antidepressants, analgesics, or anticonvulsants). These patients were treated by their dentist and referred to our university hospital because further treatment was difficult. This retrospective cohort study was conducted in accordance with the Declaration of Helsinki of the World Medical Association.

### Inclusion and exclusion criteria

Of the 30 patients with intractable IOP mentioned above, patients diagnosed with ADHD were included as the subjects in this study. Patients under 18 years of age or with impaired judgment due to severe psychosis, manic status, or depression were excluded from this study.

### Assessment and diagnosis of ADHD

All participants and their families were instructed to answer the long version of the Connors' Adult ADHD Rating Scale Self-Report (CAARS-S) or the observer-version (CAARS-O) at the time of their first medical examination in this study^29,30^. The CAARS-S and CAARS-O have eight subscales and calculate the T-scores for each. Patients with a T-score greater than 65 on the CAARS-S or CAARS-O were classified as CAARS positive, indicating a clinically significant level of ADHD symptoms. The CAARS is widely used as a measure of ADHD symptoms in adults aged ≥ 18 years.

Diagnosis of ADHD was made by a psychiatrist (S.K.) at the time of the first visit according to the criteria of the DSM-5^14^. The diagnosis was verified using the semi-structured Diagnostic Interview for ADHD in Adults 2.0 (DIVA 2.0)^31^ conducted on the first visit as well. DIVA 2.0 provides typical examples of dysfunction in the 18 diagnostic criteria for ADHD and in five domains (work/education, romantic/family relationships, social interactions, leisure/hobbies, and confidence/self-image) caused by the ADHD symptoms in daily life from childhood through adulthood, and is an aid to ADHD diagnosis. According to the DSM-5 diagnostic criteria, ADHD in a person aged > 17 years is diagnosed if at least five of the nine inattention symptoms or at least five of the nine hyperactive/impulsive symptoms are present. ADHD is classified into three types: predominantly inattentive, predominantly hyperactive-impulsive, and combined.

### Assessment of pain

Pain duration (months) was defined as the period from the onset of IOP to the patient’s first visit to our pain center. Pain intensity was evaluated using the NRS^32^, an 11-point pain rating scale, with 0 indicating no pain and 10 indicating the highest pain. The NRS assesses the maximum, minimum, and average pain intensities of patients. The minimum clinically important difference (MCID) was a −1 point (or −15.0%) decrease on the NRS. A reduction of −2 points (or −33.0%) or more on the NRS is considered an optimal improvement^33^.

### Assessment of mood state

Anxiety and depression were evaluated using the HADS^34^. The HADS is a validated assessment scale for screening psychological distress in non-psychiatric patients in clinical practice consisting of 14 questions, seven of which form a subscale for assessing anxiety (HADS-A), and the remaining seven form a subscale for assessing depression (HADS-D). All items were scored on a 4-point scale ranging from 0 to 3, with 21 points each for HADS-A and HADS-D as the highest score. HADS-A/D results of 8 points or higher on each subscale were considered clinical manifestations of anxiety and depression^35^. Based on previous studies, the MCID of the HADS was set to 1.5^36^.

### Assessment of pain catastrophizing

Catastrophic thinking associated with pain was evaluated using the PCS^37^. More intense catastrophic thinking increases the intensity of pain and daily life disability and increases the likelihood of the pain becoming chronic^38^. PCS is a self-administered questionnaire comprising 13 items; the scores for each question range from 0 to 4, with a possible total score of 0 to 52 points. A total of 30 points on the PCS corresponded to the 75^th^ percentile of PCS score distribution in patients with chronic pain. Patients with a score above the 75th percentile were at high risk of developing chronic pain^39^. MCID for PCS was between 38 and 44%^40^.

### Statistical analyses

Statistical analyses were performed using the JMP Pro version 16 (SAS Institute Japan, Tokyo, Japan). Since CAARS separates Japanese standardized groups for men and women, the CAARS subscale scores for IOP patients with ADHD were analyzed separately for men and women with the Japanese standardized group^30^. The results of the two-tailed independent samples t-test were expressed as mean differences and 95% confidence intervals (CIs). The changes in outcome variables from an individual baseline to the endpoint were analyzed using paired-sample tests. The points and percent improvement in pain NRS per dental diagnosis were evaluated using one-way analysis of variance and Tukey’s test for post-hoc analysis. The statistical significance level was set at *P* < 0.05 for convenience; however, considering the risk of type 1 error, its interpretation should be limited to that of a reference to observe the overall trend only. In addition, *P* values adjusted by the Bonferroni method are provided as corrected *P* values.

### Medication

**Figure 1:**
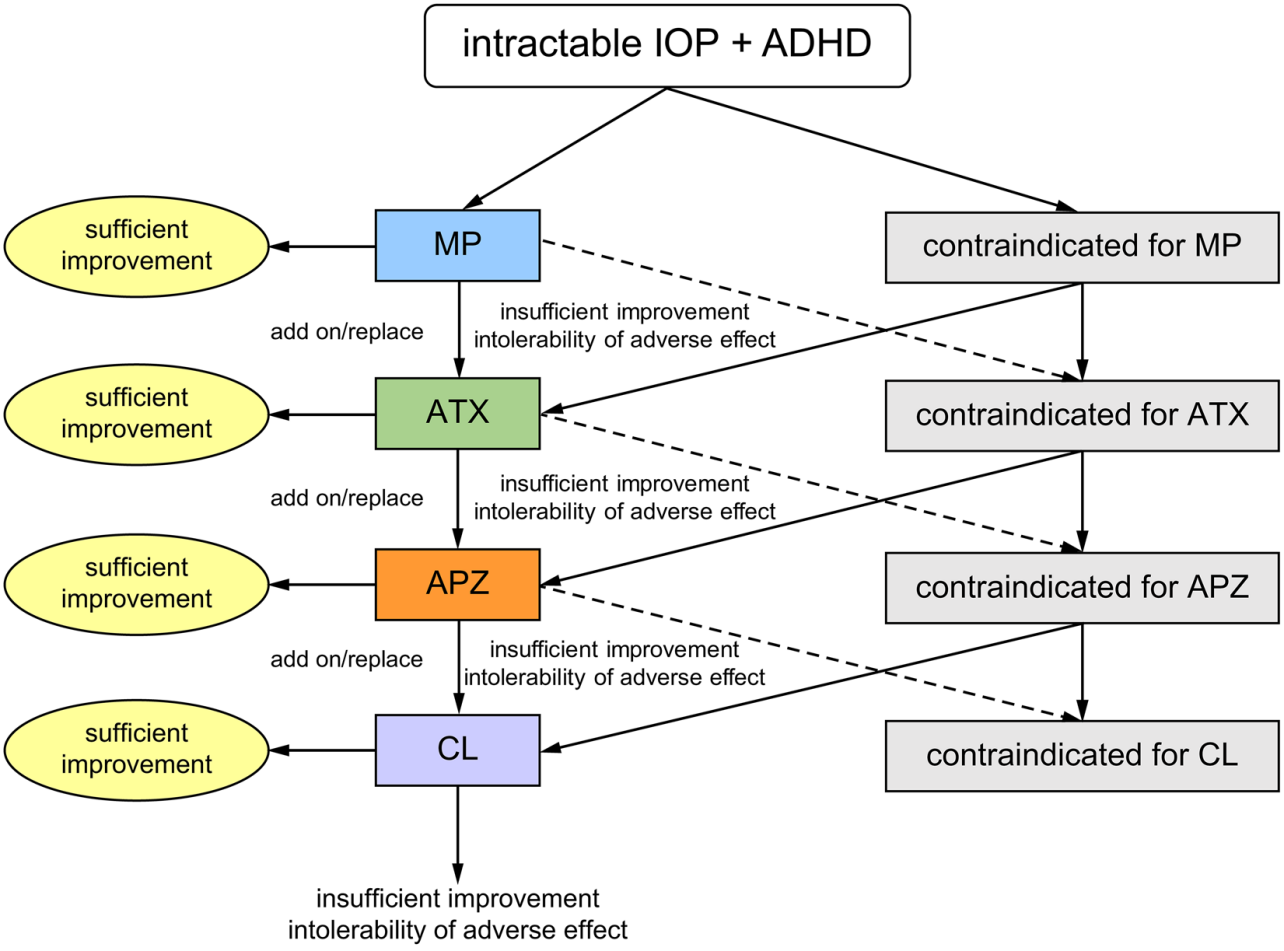
Medication algorithm for intractable IOP with ADHD. *ADHD* attention-deficit/hyperactivity disorder, *APZ* aripiprazole, *ATX* atomoxetine, *CL* clonidine, *IOP* idiopathic orofacial pain, *MP* methylphenidate.

The medication algorithm used by Dr. SK in his usual practice is shown in Fig. 1. In cases where the patient had no contraindications for the medication, the first drug of choice was the psychostimulant methylphenidate (MP)^15^. In cases where MP did not result in sufficient improvement or with intolerable adverse effects, the patient underwent combination therapy with MP and the selective norepinephrine reuptake inhibitor atomoxetine (ATX)^15^ or switched to ATX. In cases where ATX administration did not result in sufficient improvement or with intolerable adverse effects, the patient underwent combination therapy with APZ or switched to APZ. APZ is a partial agonist of dopamine D2 receptors; it is also called a dopamine system stabilizer as it suppresses excessive dopamine activity, or activates it when dopamine is underactive^15^. APZ is thought to improve ADHD^16^, chronic pain^41^, and IOP^42,43^. In cases where APZ administration did not result in sufficient improvement or with intolerable adverse effects, the patient underwent combination therapy with clonidine, a noradrenergic alpha 2 receptor agonist that is effective for ADHD^15^, or switched to clonidine. The therapeutic effects on pain NRS, HADS, and PCS were judged 2 months after the prescription was revised with sufficient improvement, in the absence of any side effects that prevented the patients from continuing the drug.

### Ethics approval and consent to participate

This study was approved by the Research Ethics Committee of Tokyo University Hospital (approval no. 3678). Informed consent was obtained orally from all subjects on the occasion of clinic visit, and all subjects provided written informed consent for participation in and publication of this study. Furthermore, the participants were guaranteed the opportunity to withdraw or refuse participation and informed that they could opt out of this study through the University of Tokyo Hospital website homepage.

## Results

### Clinical characteristics

To elucidate the clinical characteristics and treatment methods of intractable IOP with ADHD, of the 30 consecutive IOP patients referred, 25 patients with both IOP and ADHD (83.3%) were included in our analyses.

In the IOP subcategory, 14 patients had BMS, six had PIDAP, and five had PIFP. Table 1 shows the patients’ demographic and clinical characteristics. The average pain duration was 107.2 months, signifying that the patients were refractory patients who had suffered long-term pain. Among the 25 patients with a history of receiving prescription medicine, 15 (60.0%) had a history of tricyclic antidepressant use, 10 (40.0%) of clonazepam use, nine (36.0%) of pregabalin use, 19 (76.0%) of other anticonvulsant use, 10 (40.0%) of duloxetine use, two (8.0%) of potent opioid use, six (24.0%) of tramadol hydrochloride use, six (24.0%) of nonsteroidal anti-inflammatory drug use, 25 (100%) of selective serotonin reuptake inhibitor and other antidepressant use, 19 (76.0%) of atypical antipsychotic use, and 12 (48.0%) of sleeping medication use.

**Table 1:** Patient characteristics.

Variable	IOP + ADHD (N = 25)
Age, years	57.6 (15.4)
Women (n)	20 (80.0%)
Years of education	12.9 (2.9)
Presently unemployed (n)	23 (92.0%)
Married or equivalent (n)	18 (72.0%)
Pain duration, months	107.2 (102.8)
NRS maximum	7.1 (2.2)
NRS minimum	3.5 (2.6)
NRS average	6.0 (2.1)
HADS-A	8.5 (4.6)
HADS-D	9.8 (5.3)
PCS	33.7 (13.7)

*ADHD* attention-deficit/hyperactivity disorder, *HADS-A/D* Hospital Anxiety and Depression Scale-subscale for anxiety/depression, *IOP* idiopathic orofacial pain, *NRS* Numerical Rating Scale, *PCS* Pain Catastrophizing Scale.

Among the 25 patients, 19 (76.0%) had a history of psychiatric treatment, with somatic symptom disorder in nine (36.0%), depression in 10 (40.0%), anxiety disorders in five (20.0%), bipolar disorder in one (4.0%), post-traumatic stress disorder in one (4.0%), dissociation disorder in one (4.0%), and suicide attempt in one patient (4.0%). All 19 patients with a history of psychiatric treatment underwent psychiatric pharmacotherapy; however, there was no improvement in the symptoms of chronic orofacial pain.

The number of items of the diagnostic criteria for ADHD in the DSM-5 (inattention/hyperactivity-impulsivity) that each patient met is shown in Table S1 of Supplementary Information. Investigation on the ADHD subtypes indicated that three patients (12.0%) suffered from the inattentive type, two (8.0%) from the hyperactive type, and 20 (80.0%) from the combined type.

The mean scores of the eight subscales (A-H) of the CAARS-S and CAARS-O in patients with IOP are summarized in Table 2 for all patients and by sex. The comparison of CAARS scores between patients with intractable IOP with ADHD (men: 5, women: 20) and the general population in Japan (men: 245, women: 270)^30^ is also shown in Table 2, and the results are shown separately for men and women on the eight CAARS-S/O subscales, because CAARS standardization is set by gender. Since one of the criteria for the diagnosis of ADHD is that the degree of its symptoms is disproportionate to the standard developmental level, and to increase the validity of the ADHD diagnosis, we compared the CAARS scores of patients with intractable IOP with those of a standardized sample of Japanese subjects. The CAARS subscale T-scores were categorized at 5-point intervals, with T-scores of 45–55 considered “average”, 56–60 considered “slightly atypical”, 61–65 considered “mildly atypical”, 66–70 considered “moderately atypical”, and 71 or greater considered “markedly atypical”. A higher T-score for each subscale indicated more severe ADHD symptoms.

**Table 2:** Comparison of CAARS-S/O subscale scores between patients with persistent IOP and healthy controls.

Variable		All	Male						Female					
		IOP (n = 25)	IOP (n = 5)	Healthy (n = 245)	Mean difference	95% CI	*P*	Corrected *P*	IOP (n = 20)	Healthy (n = 270)	Mean difference	95% CI	*P*	Corrected *P*
		Mean (SD)	Mean (SD)	Mean (SD)					Mean (SD)	Mean (SD)				
CAARS-S	A. Inattention/memory problems	59.8 (13.0)	60.6 (11.0)	50 (10.0)	10.6	1.7–19.5	0.020	0.640	59.6 (13.7)	50 (10.0)	9.6	4.9–14.3	< 0.001	< 0.032
	B. Hyperactivity/restlessness	64.0 (14.1)	59.6 (13.5)	50 (10.0)	9.6	0.6–18.6	0.036	1.000	65.2 (14.4)	50 (10.0)	15.2	10.5–19.9	< 0.001	< 0.032
	C. Impulsivity/emotional liability	53.2 (11.5)	49.8 (8.2)	50 (10.0)	−0.2	−9.1 to 8.7	0.965	1.000	54.1 (12.2)	50 (10.0)	4.1	−0.5 to 8.7	0.083	1.000
	D. Problems with self-concept	58.4 (9.4)	61.0 (7.3)	50 (10.0)	11.0	2.2–19.9	0.015	0.480	57.8 (9.9)	50 (10.0)	7.8	3.2–12.4	0.001	0.032
	E. DSM-IV Inattentive Symptoms	61.3 (14.2)	57.4 (10.9)	50 (10.0)	7.4	−1.5 to 16.3	0.100	1.000	62.3 (15.0)	50 (10.0)	12.3	7.6–17.0	< 0.001	< 0.032
	F. DSM-IV hyperactive-impulsive symptoms	63.2 (13.0)	63.4 (10.7)	50 (10.0)	13.4	4.5–22.3	0.003	0.096	63.1 (13.8)	50 (10.0)	13.1	8.4–17.8	< 0.001	< 0.032
	G. DSM-IV ADHD symptoms total	63.3 (12.8)	62.0 (10.4)	50 (10.0)	12.0	3.1–20.9	0.008	0.256	63.6 (13.5)	50 (10.0)	13.6	8.9–18.3	< 0.001	< 0.032
	H. ADHD index	61.8 (11.5)	60.2 (5.7)	50 (10.0)	10.2	1.4–19.0	0.024	0.768	62.2 (12.7)	50 (10.0)	12.2	7.5–16.9	< 0.001	< 0.032
CAARS-O	A. Inattention/memory problems	65.4 (12.4)	77.2 (5.3)	50 (10.0)	27.2	18.4–36.0	< 0.001	< 0.032	62.4 (12.0)	50 (10.0)	12.4	7.8–17.0	< 0.001	< 0.032
	B. Hyperactivity/restlessness	65.5 (12.2)	74.8 (10.2)	50 (10.0)	24.8	15.9–33.7	< 0.001	< 0.032	63.2 (11.8)	50 (10.0)	13.2	8.6–17.8	< 0.001	< 0.032
	C. Impulsivity/emotional liability	65.0 (15.3)	71.6 (11.7)	50 (10.0)	21.6	12.7–30.5	< 0.001	< 0.032	63.4 (15.9)	50 (10.0)	13.4	8.6–18.2	< 0.001	< 0.032
	D. Problems with self-concept	65.2 (14.4)	71.2 (14.5)	50 (10.0)	21.2	12.2–30.2	< 0.001	< 0.032	63.8 (14.4)	50 (10.0)	13.8	9.1–18.5	< 0.001	< 0.032
	E. DSM-IV Inattentive symptoms	68.0 (16.1)	84.4 (8.8)	50 (10.0)	34.4	25.5–43.3	< 0.001	< 0.032	63.9 (14.9)	50 (10.0)	13.9	9.2–18.6	< 0.001	< 0.032
	F. DSM-IV hyperactive-impulsive symptoms	63.4 (17.0)	79.2 (15.6)	50 (10.0)	29.2	20.2–38.2	< 0.001	< 0.032	59.4 (15.2)	50 (10.0)	9.4	4.6–14.2	< 0.001	< 0.032
	G. DSM-IV ADHD symptoms total	66.5 (15.8)	83.4 (10.9)	50 (10.0)	33.4	24.5–42.3	< 0.001	< 0.032	62.3 (14.0)	50 (10.0)	12.3	7.6–17.0	< 0.001	< 0.032
	H. ADHD index	70.9 (12.0)	81.0 (4.4)	50 (10.0)	31.0	22.2–39.8	< 0.001	< 0.032	68.4 (12.0)	50 (10.0)	18.4	13.8–23.0	< 0.001	< 0.032

*ADHD* attention-deficit/hyperactivity disorder, *CAARS-O* Conners’ Adult ADHD Rating Scale observer-version, *CAARS-S* Conners’ Adult ADHD Rating Scale self-report, *CI* confidence interval, *DSM-IV* Diagnostic and Statistical Manual of Mental Disorders, fourth edition, *IOP* Idiopathic orofacial pain, *SD* standard deviation.

P values were corrected by multiplying 32, the number of tests for multiple testing within the table and expressed as corrected p (i.e., Bonferroni correction).

Among the eight subscales of the CAARS, subscales E, F, and G assess the extent to which a patient's ADHD symptoms meet the criteria of DSM-IV; subscale H, called the ADHD index, indicates the extent to which a patient's ADHD symptoms require treatment. Except for the CAARS-S subscale E for men, which was not significantly different from the standardized sample, both male and female patients with intractable IOP in this study had significantly higher scores on subscales E, F, and G on both CAARS-S/O compared with standardized samples. On subscale H of the CAARS-S/O, both male and female patients with intractable IOP scored significantly higher than the standardized sample on the CAARS-S/O.

Furthermore, although not shown in Table 2, among the eight subscales of the CAARS-S/O for patients, male patients had significantly higher scores than female patients on CAARS-O subscale A (mean difference: 14.8; CI, 3.3–26.3; *P* = 0.01), CAARS-O subscale E (mean difference: 20.6; CI, 6.0–35.1; *P* = 0.008), CAARS-O subscale F (mean difference: 19.8; CI, 4.0–35.6; *P* = 0.02), CAARS-O subscale G (mean difference: 21.2; CI, 7.2–35.1; *P* = 0.004), and CAARS-O subscale H (mean difference: 12.6; CI, 1.1–24.1; *P* = 0.03).

### Medication regimen and outcomes

Five of the 25 patients with IOP diagnosed with ADHD did not undergo pharmacotherapy (patients who did not wish to receive pharmacotherapy received outpatient cognitive behavioral therapy with Dr. SK as an alternative protocol). The changes in the pain scale scores before and after treatment for the 20 patients (BMS: 12, PIFP: 5, PIDAP: 3) who underwent pharmacotherapy according to the treatment regimen used in this study are shown in Table 3. In the treatment group, the maximum, minimum, and average pain NRS improved by 2.8 ± 2.5, 1.6 ± 2.6, and 2.7 ± 2.6 points, respectively. PCS showed an improvement of 13.1 ± 14.8 points, indicating statistically significant differences.

**Table 3:** Comparison of the degree of improvement in each evaluation scale before and after treatment.

Treatment group (N = 20)	Baseline	Post treatment	Mean difference	CI (95%)	t	*P*	Corrected *P*
NRS maximum	6.8 (2.3)	4.0 (2.9)	2.8	1.6 to 4.0	5.04	< 0.001	< 0.006
NRS minimum	3.1 (2.4)	1.6 (2.0)	1.6	0.3 to 2.8	2.70	0.014	0.084
NRS average	5.7 (2.1)	3.0 (2.6)	2.7	1.5 to 3.9	4.65	< 0.001	< 0.006
HADS-A	8.1 (4.9)	6.5 (4.9)	1.6	−1.0 to 4.2	1.29	0.211	1.000
HADS-D	9.4 (5.7)	7.4 (5.5)	2.0	−0.4 to 4.4	1.74	0.097	0.582
PCS	33.2 (12.8)	20.1 (13.7)	13.1	6.0 to 20.3	3.85	0.001	0.006

*CI* confidence interval, *HADS-A/D* Hospital Anxiety and Depression Scale-subscale for anxiety/depression, *NRS* numerical rating scales, *PCS* Pain Catastrophizing Scale.

P values were corrected by multiplying with 6, the number of tests for multiple testing within the table, and expressed as corrected p (i.e., Bonferroni correction).

The administered dose of each medication and the percentage improvement in the average NRS for pain are shown in Table S1 of Supplementary Information. The average dose of MP monotherapy was 39.6 ± 19.7 mg/day, with a 41.7 ± 45.0% improvement. The average doses of the combination of MP and ATX were 57.0 ± 26.0 mg/day and 76.7 ± 45.1 mg/day, respectively, with a 46.7 ± 61.1% improvement. The average doses of the combination of ATX and APZ were 72 mg/day and 6 mg/day, respectively, with a 100 ± 0.0% improvement. The average dose of ATX monotherapy was 110.0 ± 20.0 mg/day, with a 53.1 ± 22.6% improvement. The doses of the combination of ATX and APZ were 80.0 mg/day and 6.0 mg/day, respectively, with a 100 ± 0.0% improvement. The APZ monotherapy dose was 7.5 ± 3.9 mg/day, with 68.3 ± 16.9% improvement. The CL monotherapy dose was 300.0 mg/day, with a 20.0 ± 0.0% improvement. There were no significant differences in the NRS improvement scores or percentages according to sex, age, or medication. When investigated using the dental diagnosis (BMS, PIDAP, and PIFP), one-way analysis of variance showed no significant difference in the number of improved NRS points (BMS: 2.7 ± 2.8, PIDAP: 0.0 ± 1.0, PIFP: 4.4 ± 0.9) (F(2,19) = 3.36 *P* = 0.059); however, there was a significant difference in the percentage of improved NRS (BMS: 50.7 ± 38.9%, PIDAP: −1.9 ± 17.2%, PIFP: 79.8 ± 23.2%) (F(2,19) = 5.50 *P* < 0.05), and post-hoc analysis (Tukey’s test) showed a significant difference in PIDAP vs. PIFP (*P* < 0.05).

## Discussion

This study demonstrated that a high percentage (83.3%) of the 30 patients with intractable IOP had ADHD, and the CAARS-S/O DSM and ADHD index scores for patients with refractory orofacial pain were higher than those for the general population in almost all cases. Moreover, the pharmacotherapy used in this study, consisting of ADHD medication and a dopamine system stabilizer, may result in significant clinical improvements in pain, anxiety, depression, and pain catastrophizing.

### ADHD comorbidity rate

In this study, 25 of the 30 patients with refractory orofacial pain (83.3%; women: 20) were diagnosed with ADHD. To the best of our knowledge, no previous surveys have investigated the incidence of ADHD in patients with IOP. This study revealed that ADHD occurs frequently in patients with refractory IOP. Therefore, we believe that the results of the present study provide a new perspective on the clinical care of and research on patients with IOP. However, given the specific referral source and small sample size in this study, the rate of ADHD comorbidity in patients with intractable IOP should only be viewed as an indication of the overall trend.

A study of 153 patients with chronic pain, including lower back pain and widespread pain, reported the coexistence of ADHD in 72.5% of the cases^44^. In addition, Young et al. reported that 80% of patients with fibromyalgia had ADHD^19^. The findings reported in these studies are similar to that of the present study. In contrast, other studies^17,21^ reported that the incidences of ADHD among patients with fibromyalgia were 25% and 29.5%, respectively, indicating large discrepancies between the studies. There are cases in which the patient does not recognize the symptoms of ADHD; thus, assessments by family members and other third parties are important. However, in a previous survey of ADHD on patients with fibromyalgia, the Wender Utah Rating Scale^45^ or the Adult ADHD Self-Report Scale-V1.1^46^ was used, and in both cases, only the patient himself/herself was expected to answer the questionnaire. In contrast, the CAARS used in this study instructed the patients and their families to answer the questionnaire. To ensure the accurate clinical assessment of ADHD, it is important to obtain information from multiple sources, and it is preferable to collect information from both self-reports by the patient and observer reports by family members and other third parties^29^. It is preferable to refer to the findings obtained from family members on the CAARS-O when diagnosing ADHD using the structured interview of the Conners’ Adult ADHD Diagnostic Interview for DSM-IV^47^. Furthermore, in clinical interviews, it is easier to extract information that contributes to the diagnosis by asking the patient questions by referring to the answers to each CAARS question by the patient and his/her family^29^. Therefore, we believe that it is important to screen for and diagnose ADHD using both the CAARS-S and CAARS-O.

The ADHD subtypes identified in the present study were inattentive (12.0%), hyperactive (8.0%), and combined (80.0%). Although there are no reports on ADHD subtypes in adults in the general population, surveys on children reported the ratio of inattentive: hyperactive-impulsive: combined as 3.5: 1.3: 2.2^48,49^. A study on ADHD subtypes comorbid with fibromyalgia in adults reported that approximately one-third of the cases were inattentive and about two-thirds were combined^50^. Therefore, comorbidities with chronic pain may more likely present in the combined subtype than in other ADHD subtypes. Based on our experience, the characteristic feature of ADHD in patients with IOP is difficulty in continuing with time-consuming treatment; that is, they tend to opt for risky treatments and on not experiencing improvement immediately, they tend to go from doctor to doctor, displaying impulsive behavior. The symptoms of inattention are being prone to derailed and uncoordinated conversations.

### Severity as shown by the ADHD scale CAARS score

Among our 25 patients, 16 (64.0%) screened positive for both the CAARS-S/O, 23 (92.0%) screened positive for the CAARS-S or the CAARS-O, and two (8.0%) screened negative for both. Among all patients, only one had been previously diagnosed with ADHD. However, even when using the stricter criteria for a positive result on the CAARS-S and CAARS-O, 64.0% of the patients tested positive for ADHD, indicating that screening for adult ADHD is required when examining and treating patients with refractory IOP.

Except for CAARS-S subscale E for men, both men and women with intractable IOP in this study had significantly higher scores for subscale E (DSM-IV Inattentive Symptoms) and subscale F (DSM-IV Hyperactive-Impulsive Symptoms) on both CAARS-S/O than the standardized sample. Thus, men and women with intractable IOP experienced both inattention and hyperactivity-impulsivity symptoms at a greater intensity than the standardized sample did. This is consistent with the finding that 80% of the patients in this study had combined type ADHD.

In terms of subscale H, both men and women scored significantly higher than the standardized sample on both the CAARS-S/O. The CAARS-O subscale H, that is, the ADHD index, exceeded a T-score of 65 for both men and women; therefore, the ADHD symptoms of patients with refractory IOP from the perspective of the family were considered to be at the level requiring psychiatric treatment.

Comprehensive observation of the eight subscales of the CAARS-S/O indicates that in the CAARS-O, both men and women with intractable IOP scored significantly higher on all eight subscales than the standardized sample. When we examined CAARS-O scores for all 25 patients with IOP (males and females), we found that on all scales except for the F scale, the T-score was > 65, and ADHD symptoms in patients with IOP from the perspective of the family were at the clinical psychiatric level.

### Effectiveness of treatment

The optimal MCID for the degree of improvement in chronic pain NRS was 33% on an average^33^. Among the 20 patients in the present study who underwent pharmacotherapy, pain improved by an average of 47.4%, indicating that the degree of pain improvement exceeded the optimal MCID. The MCID for HADS-A/D is 1.5 points^36^. In the present study, the HADS-A and HADS-D did not show statistically significant differences in the before vs. after treatment comparison; however, HADS-A showed an improvement of 1.6 points and HADS-D showed an improvement of 2 points, indicating that both achieved MCID. The MCID for PCS has been reported to improve by 38%^40^. In the present study, the PCS of patients who underwent pharmacotherapy improved by 39.5%, indicating that MCID was attained. The above findings suggest that the pharmacotherapies in the present study that utilized ADHD medication and the dopamine system stabilizer APZ are capable of achieving clinically significant improvement in refractory IOP with ADHD and symptoms related to pain such as anxiety and depression.

The pain NRS improvement % of patients with PIDAP was significantly lower than that of those with PIFP. The improvement in pain NRS in the three IOPs could be due to the following reasons: first, PIFP does not require dental procedures to be undertaken, whereas dental procedures in PIDAP tend to make treatment more difficult iatrogenically; this may explain the difference in the improvement between the two groups. Second, the number of patients with PIDAP was small (only three), and although PIDAP in patients No. 5 and 6 showed significant clinical improvement, the patients were unaware of their improvement, which was not reflected in the improvement of their pain NRS scores.

Young reported that chronic pain is associated with ADHD-related attention deficit, and that improving attention with ADHD treatments could activate the inhibitory filtering system and improve pain^51^. Stimulants used as ADHD medication have been reported to improve the pain symptoms of fibromyalgia^20,52^, and ATX improves the cognitive disorder of fibromyalgia known as “fibro-fog”^15^ and atypical odontalgia^53^. In addition, ADHD medications (MP and/or ATX) have been reported to improve average pain NRS associated with chronic pain in patients suspected of somatic symptom disorder by 3.5 ± 2.1 points^44^.

APZ is an atypical antipsychotic that exhibits properties of a partial agonist of Dopamine D2 and serotonin 5-hydroxytryptamine (5-HT) 1A receptors and a potent antagonist of 5-HT 2A receptors, resulting in enhanced dopamine neurotransmission^15^. Theoretically, low phasic dopamine activity is associated with increased pain through the decreased release of μ-opioids^54^; therefore, APZ could have improved IOP by activating phasic dopaminergic transmission resulting in the release of μ-opioids.

Clonidine is a partial agonist of noradrenergic α2 receptors, and its action on α2 receptors in the spinal cord and peripheral nerves produces analgesia comparable with or better than that of acetaminophen^55^. Hence, it has been applied in pain management via intravenous and intrathecal administration in the perioperative period^56^. However, no previous studies have reported the effects of orally administered clonidine on chronic primary pain, and this is the first report of such a study. Furthermore, α2 receptors are abundantly distributed in the prefrontal cortex, and clonidine is regarded as an ADHD drug, as it can improve inattention and hyperactivity/impulsivity^15^. Therefore, clonidine is a potential treatment option for patients with IOP comorbid with ADHD, especially those with elevated blood pressure. Patient No. 18 in this study was hypertensive; 300 mg of clonidine improved her BMS, ADHD, and hypertension.

### Hypothesis on IOP and ADHD comorbidity and treatment mechanisms

IOP, as with other chronic primary pain syndromes, is caused by central sensitization due to central nervous system dysfunction^57^. Dopaminergic and prefrontal dysfunction based on ADHD can cause such central sensitization^58^. Dopamine plays a central role in pain perception and the descending pain suppression pathways, and reduced dopamine levels may increase pain^59^. ADHD is also assumed to have dopaminergic dysfunction^28^ and is a vulnerability to chronic pain. The prefrontal cortex is also functionally coupled to the descending pain inhibitory pathways and can act as a virtual filter to reduce unpleasant stimuli, such as pain and itching^51,60^. The prefrontal cortex performance follows an inverted U-shaped curve with respect to dopamine and noradrenaline activation and is maximized when the concentrations of both transmitters are moderate^61^. However, since ADHD assumes impaired dopamine and noradrenaline neurotransmission as pathophysiology, this filter does not function adequately, and ADHD is thought to be vulnerable to pain. As shown in this study, ADHD medications and the dopamine system stabilizer may activate the descending inhibitory system and reduce pain by moderately modulating the dopamine and noradrenaline transmission in the reward system and prefrontal cortex.

TCAs are effective in the treatment of IOP^9^ due to their serotonin and noradrenaline reuptake inhibitory effects^15^. However, TCAs increase the dopamine and noradrenaline levels in the prefrontal cortex, since there are few dopamine transporters in the prefrontal cortex and there is a reuptake of dopamine by the noradrenaline transporter^15^. Thus, TCAs can improve ADHD symptoms to the same degree as ADHD medications^62^. If the higher comorbidity of ADHD and intractable IOP in the present study can be generalized to IOP in general, the following hypotheses can be considered: the effectiveness of TCAs for IOP may be due in part to TCAs improving ADHD comorbid with IOP by improving dopamine and noradrenaline neurotransmission, similar to the ADHD medications and the dopamine system stabilizer used in this study.

This study has two limitations. First, the participants were patients who were referred by a dentist at a tertiary medical center. The participants were special cases in which treatment was difficult even when they underwent specialized treatment, and the sample size was small; therefore, caution is required when attempting to apply the results of this study to patients with IOP treated at general dental clinics. Second, the therapeutic effect in the present study cannot be attributed simplistically to the ADHD medication or APZ. Because the participants in this study were often taking antidepressants and analgesics, there is a possibility that the interaction of these drugs with ADHD treatments and APZ provided improvement. Therefore, care should be taken when interpreting the results of this study.

## Conclusions

This study reported on persistent IOP with ADHD comorbidity. But as mentioned above, it has been reported that ADHD is also associated with chronic primary pain such as fibromyalgia, migraine, and chronic back pain. Further, chronic primary pain is associated with extreme emotional distress, such as anxiety, depression, and anger/frustration, and is also associated with functional disabilities in activities of daily living and decreased participation in social roles^26^. The neurocognitive traits of ADHD may be responsible for these emotional disorders and dysfunction in activities of daily living.

In the future, when examining and treating fibromyalgia, migraine, chronic low back pain, IOP, and other types of chronic primary pain, ADHD screening should be conducted, and effective pharmacotherapies should be considered, provided the patient satisfies the diagnostic criteria. There remains a need to further study the relationship between chronic pain and ADHD by conducting surveys in more common clinical settings and controlled interventional studies.

## Supplementary Information

Supplementary Table S1.

## Data Availability

The datasets used and/or analysed during the current study are available from the corresponding author on reasonable request.

## Abbreviations

5-HT: 5-Hydroxytryptamine
ADHD: Attention-deficit/hyperactivity disorder
APZ: Aripiprazole
ATX: Atomoxetine
BMS: Burning mouth syndrome
CAARS-O: Connors' Adult ADHD Rating Scale Observer-version
CAARS-S: Connors' Adult ADHD Rating Scale Self-Report
CI: Confidence interval
DIVA: Diagnostic Interview for ADHD in Adults
DSM-5: Diagnostic and Statistical Manual of Mental Disorders-5
HADS: Hospital Anxiety and Depression Scale
HADS-A: Hospital Anxiety and Depression Scale, subscale for assessing anxiety
HADS-D: Hospital Anxiety and Depression Scale, subscale for assessing depression
ICOP: International Classification of Orofacial Pain
IOP: Idiopathic orofacial pain
MCID: Minimum clinically important difference
MP: Methylphenidate
NRS: Numerical Rating Scale
PCS: Pain Catastrophizing Scale
PIDAP: Persistent idiopathic dentoalveolar pain
PIFP: Persistent idiopathic facial pain
TCAs: Tricyclic antidepressants
