# Supplementary material for: Diagnosis and treatment of intractable idiopathic orofacial pain with attention-deficit/hyperactivity disorder
Source: Sci Rep. 2023 Jan 30;13:1678. doi: 10.1038/s41598-023-28931-3 (PMC9887013; doi:10.1038/s41598-023-28931-3)
Supplement: Supplementary file 1 — Supplementary Table S1. [file 41598_2023_28931_MOESM1_ESM.docx]

**Table S1**. Details of ADHD diagnosis in patients with IOP: number of items in ADHD diagnostic criteria, history of psychiatric treatment, content of drug therapy, and degree of improvement in pain.

| No. | Sex | Age  (years) | Dental diagnosis | Symptoms of attention-deficit | Symptoms of hyperactivity-impulsivity | ADHD subtype | Prior psychiatric condition | Medication | Medication regimen | | | | Baseline NRS | Post treatment NRS | Improvement in NRS (%) |
| --- | --- | --- | --- | --- | --- | --- | --- | --- | --- | --- | --- | --- | --- | --- | --- |
|  |  |  |  |  |  |  |  |  | MP (mg/day) | ATX (mg/day) | APZ (mg/day) | CL (μg/day) |  |  |  |
| 1 | F | 31 | PIFP | 5 | 4 | Inattentive | Anxiety disorder, somatic symptom disorder | + | 18 |  |  |  | 5 | 0 | 100.0 |
| 2 | F | 58 | PIDAP | 7 | 9 | Combined | Bipolar disorder | + | 54 |  |  |  | 7 | 6 | 14.3 |
| 3 | F | 68 | BMS | 9 | 7 | Combined | Depression | + | 54 |  |  |  | 7 | 6 | 14.3 |
| 4 | F | 71 | BMS | 9 | 9 | Combined | Depression | + | 18 |  |  |  | 5 | 1 | 80.0 |
| 5 | M | 67 | PIDAP | 6 | 7 | Combined |  | + | 54 |  |  |  | 6 | 6 | 0.0 |
| 6 | F | 34 | PIDAP | 9 | 6 | Combined | Depression, adhd | + | 72 | 120 |  |  | 5 | 6 | -20.0 |
| 7 | F | 37 | BMS | 9 | 7 | Combined | Depression, post traumatic stress disorder | + | 27 | 30 |  |  | 5 | 2 | 60.0 |
| 8 | M | 58 | BMS | 7 | 5 | Combined | Depression | + | 72 | 80 |  |  | 1 | 0 | 100.0 |
| 9 | F | 66 | PIFP | 8 | 8 | Combined | Depression, somatic symptom disorder | + | 72 |  | 6 |  | 3 | 0 | 100.0 |
| 10 | F | 62 | PIFP | 9 | 9 | Combined | Depression, dissociation disorder | + |  | 120 |  |  | 7 | 2 | 71.4 |
| 11 | F | 72 | PIFP | 6 | 8 | Combined | Somatic symptom disorder | + |  | 120 |  |  | 9 | 5 | 44.4 |
| 12 | F | 75 | BMS | 7 | 7 | Combined | Anxiety disorder, somatic symptom disorder | + |  | 80 |  |  | 8 | 6 | 25.0 |
| 13 | M | 80 | BMS | 8 | 7 | Combined |  | + |  | 120 |  |  | 7 | 2 | 71.4 |
| 14 | F | 74 | BMS | 5 | 3 | Inattentive | Somatic symptom disorder | + |  | 80 | 6 |  | 9 | 0 | 100.0 |
| 15 | F | 50 | BMS | 8 | 5 | Combined |  | + |  |  | 3 |  | 2 | 1 | 50.0 |
| 16 | F | 56 | BMS | 5 | 6 | Combined |  | + |  |  | 9 |  | 7 | 2 | 71.4 |
| 17 | M | 37 | PIFP | 8 | 9 | Combined | Somatic symptom disorder | + |  |  | 12 |  | 6 | 1 | 83.3 |
| 18 | F | 68 | BMS | 8 | 1 | Inattentive |  | + |  |  |  | 300 | 5 | 4 | 20.0 |
| 19 | F | 62 | BMS | 9 | 7 | Combined |  | + |  |  |  |  | 4 | 2 | 50.0 |
| 20 | F | 68 | BMS | 1 | 8 | Hyperactive-impulsive | Depression, suicide attempt | + |  |  |  |  | 6 | 8 | -33.3 |
| 21 | F | 41 | PIDAP | 9 | 9 | Combined | Anxiety disorder, somatic symptom disorder | - |  |  |  |  | 7 | 7 | 0.0 |
| 22 | F | 54 | BMS | 4 | 6 | Hyperactive-impulsive | Depression, somatic symptom disorder | - |  |  |  |  | 7 | 8 | -14.3 |
| 23 | F | 58 | PIDAP | 8 | 8 | Combined | Anxiety disorder | - |  |  |  |  | 10 | 9 | 10.0 |
| 24 | F | 70 | BMS | 8 | 8 | Combined | Depression | - |  |  |  |  | 7 | 8 | -14.3 |
| 25 | M | 24 | PIDAP | 9 | 6 | Combined | Anxiety disorder, somatic symptom disorder | - |  |  |  |  | 6 | 8 | -33.3 |

ADHD, Attention-deficit hyperactivity disorder; APZ, aripiprazole; ATX, atomoxetine; BMS, burning mouth syndrome; CL, clonidine; IOP, idiopathic orofacial pain; MP, methylphenidate; NRS, numerical rating scales; PIDAP, persistent idiopathic dentoalveolar pain; PIFP, persistent idiopathic facial pain
